# Supplementary material for: Neto proteins differentially modulate the gating properties of Drosophila NMJ glutamate receptors
Source: bioRxiv. 2024 Apr 26:2024.04.22.590603. Preprint. [Version 1] doi: 10.1101/2024.04.22.590603 (PMC11188076; doi:10.1101/2024.04.22.590603)
Supplement: Supplement 1 [file NIHPP2024.04.22.590603v1-supplement-1.pdf]

bioRxiv preprint doi: https://doi.org/10.1101/2024.04.22.590603; this version posted April 26, 2024. The copyright holder for this preprint (which was not certified by peer review) is the author/funder. This article is a US Government work. It is not subject to copyright under 17 USC 105 and is also made available for use under a CC0 license.

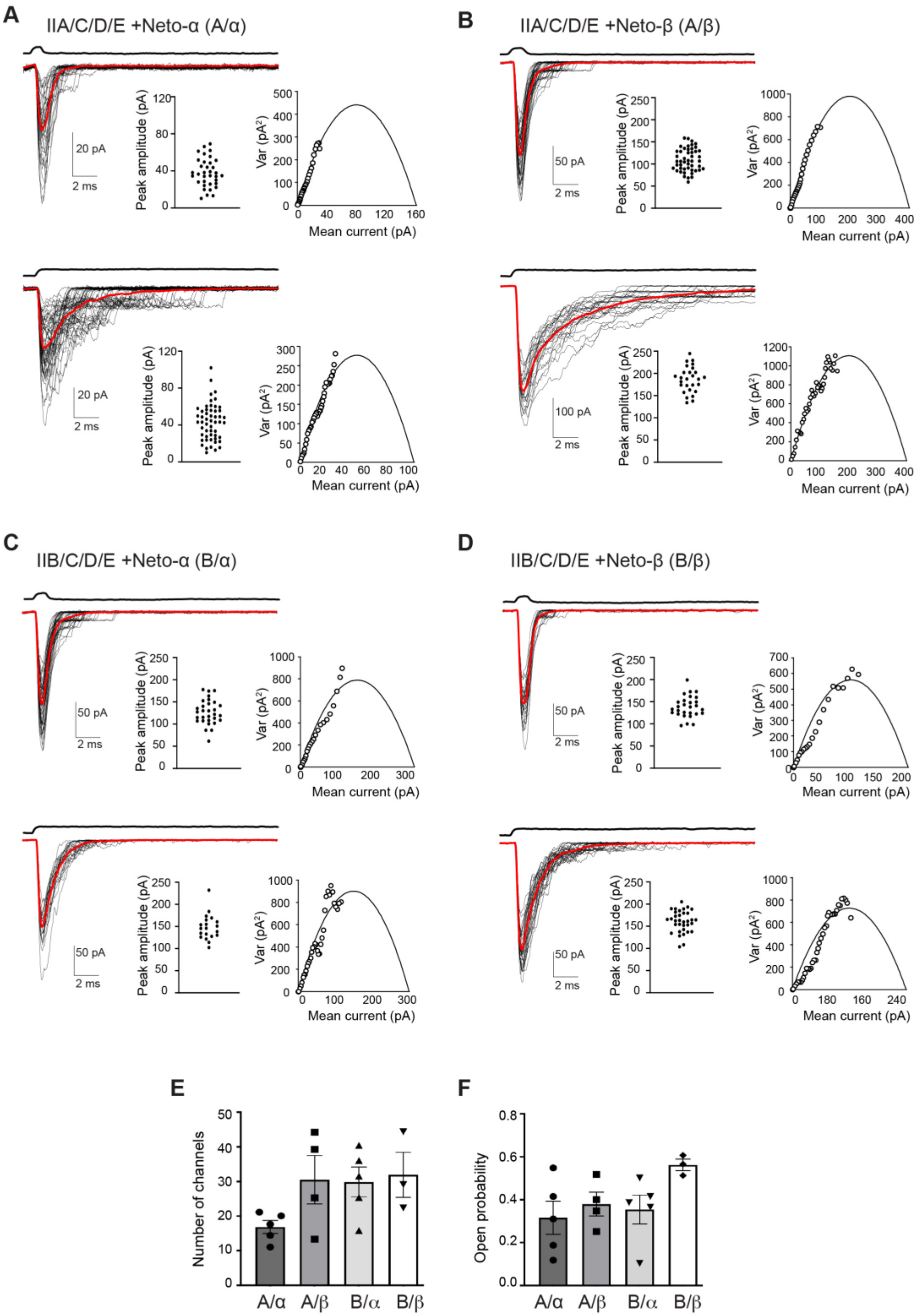

bioRxiv preprint doi: https://doi.org/10.1101/2024.04.22.590603; this version posted April 26, 2024. The copyright holder for this preprint (which was not certified by peer review) is the author/funder. This article is a US Government work. It is not subject to copyright under 17 USC 105 and is also made available for use under a CC0 license.

Supplemental figure S1. Nonstationary analysis of variance of responses to 10 mM glutamate applied for 1 ms (upper traces) and 100 ms (lower traces) to outside-out patches from HEK cells transfected with various iGluR/Neto receptor complexes as indicated.

(**A-D**) Superimposed individual responses are shown in black; the average current in red; open tip junction currents measured at the end of the experiments are shown at the top. The holding potential was - 60mV for all recordings. The insets in each panel show the peak amplitude for all trials (left) and the current-variance relationship (right) fit with the function $\sigma^2=iI - I^2/N$, where σ^2 is the variance, i is the mean current, N is the number of channels. (**E**) The mean number of channels and the open probability (**F**) determined by variance analyses of responses for 3-5 patches for different iGluR/Neto channel complexes as indicated, showing responses for individual patches and the mean ± SEM.

bioRxiv preprint doi: https://doi.org/10.1101/2024.04.22.590603; this version posted April 26, 2024. The copyright holder for this preprint (which was not certified by peer review) is the author/funder. This article is a US Government work. It is not subject to copyright under 17 USC 105 and is also made available for use under a CC0 license.

Recombinant iGluRs	Closed time (ms)	Open time (ms)	Burst length
A/α	Bimodal	0.74, 3.75	0.76, 3.12
A/α + Con A	0.6, 3.8	0.67, 7.53	0.64, 11.53
A/β	Bimodal	1.11, 4.51	1.28, 4.38
A/β + Con A	Bimodal	5.60, > 100 *	3.32, > 100 **

* Openings of duration in excess of 100 ms were observed

** Bursts of duration in excess of 100 ms were observed

Supplemental Table 1. Analysis of single channel kinetics for recombinant type A/α and A/β receptor complexes for control patches, and patches obtained from HEK cells treated with Con A. Closed time histograms revealed bimodal distributions for all conditions, but except for A/α + Con A the number of events was too small to allow an accurate estimate of the lifetime. Log binned open time and burst length distributions were fit with the sum of two exponentials as shown below (Figure 3). For receptor complexes with Con A we observed single openings and bursts of openings which exceeded the length of the 100 ms application of glutamate. Examination of the lifetime of openings in a burst revealed longer openings for each subconductance state after application of Con A for both A/α and A/β (Supplemental figure S2).

Native iGluRs	Closed time ms	Open time ms	Burst Length
Nishikawa 1995	Tau crit 1 ms	NR	0.069, 0.9, 4.9
Broadie 1993	NR	0.23, 2.1	NR
Heckmann 1995	0.073, 0.661	0.071, 1.81	NR

1. Nishikawa, Y. Kidokoro, Junctional and extrajunctional glutamate receptor channels in *Drosophila* embryos and larvae. *J Neurosci* **15**, 7905-7915 (1995).
2. K. S. Broadie, M. Bate, Development of the embryonic neuromuscular synapse of *Drosophila melanogaster*. *J Neurosci* **13**, 144-166 (1993).
3. M. Heckmann, J. Dudel, Recordings of glutamate-gated ion channels in outside-out patches from Drosophila larval muscle. *Neuroscience letters* **196**, 53-56 (1995).

Supplemental Table 2. Analysis of single channel kinetics reported in prior studies on native extrajunctional receptors expressed in *Drosophila* muscle. NR indicates Not Reported

bioRxiv preprint doi: https://doi.org/10.1101/2024.04.22.590603; this version posted April 26, 2024. The copyright holder for this preprint (which was not certified by peer review) is the author/funder. This article is a US Government work. It is not subject to copyright under 17 USC 105 and is also made available for use under a CC0 license.

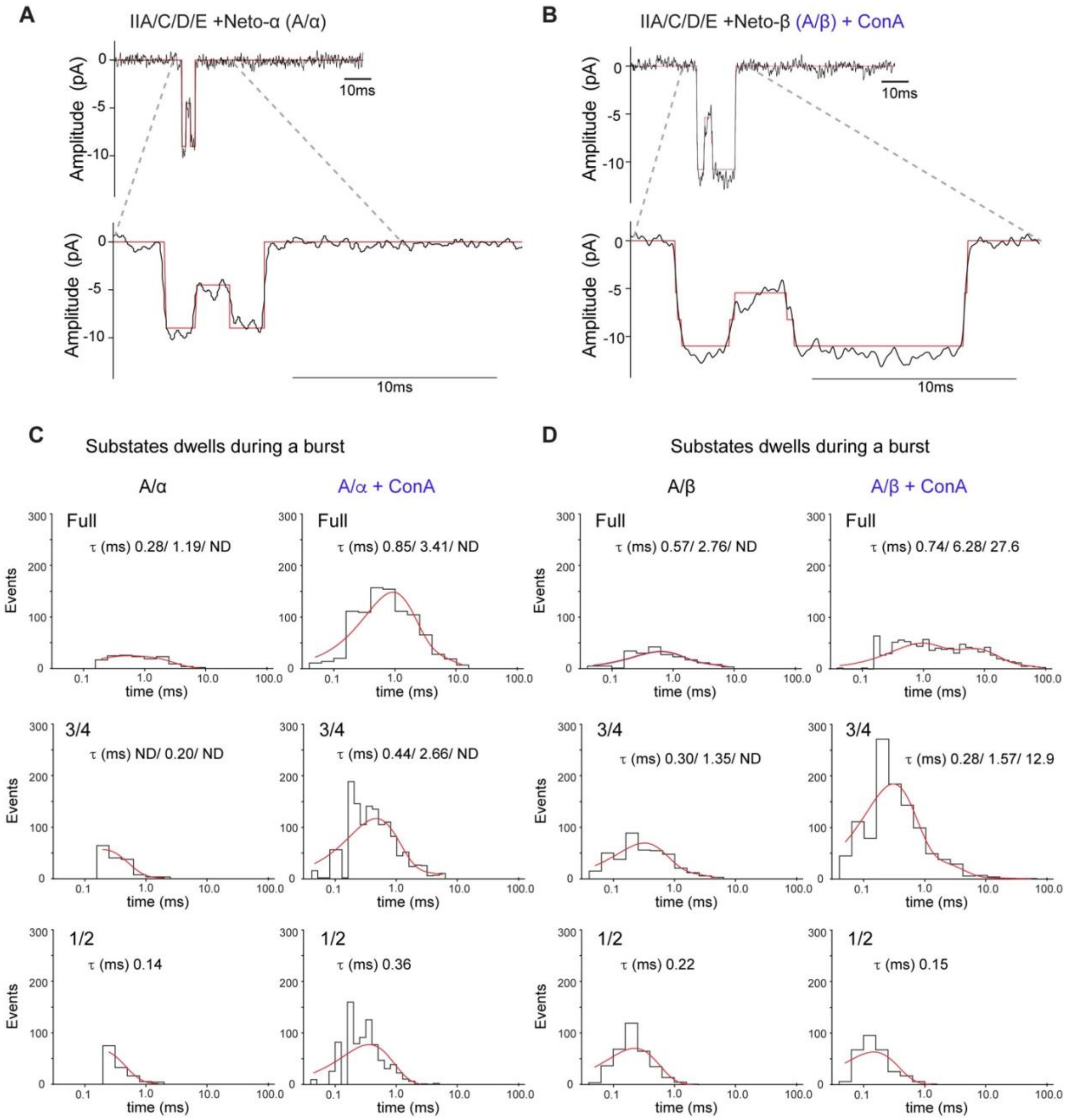

Supplemental figure S2. Modulation of substate activity by Con A.

(**A-B**) Representative responses to 10 mM glutamate applied for 100 ms to outside-out patches from HEK cells transfected with A/α and A/β receptor complexes, as indicated. The idealized traces (in red) show transitions from the fully open state to the ½ level. (**C-D**) Dwell time histograms for substate occupancy during a burst for control patches (left panels), and for patches from HEK cells treated with Con A (right panels) for A/α (**C**) and A/β (**D**), fit with the sum of 1-3 exponentials of fast, intermediate and slow time constants, as indicated. ND- not detected.

bioRxiv preprint doi: https://doi.org/10.1101/2024.04.22.590603; this version posted April 26, 2024. The copyright holder for this preprint (which was not certified by peer review) is the author/funder. This article is a US Government work. It is not subject to copyright under 17 USC 105 and is also made available for use under a CC0 license.

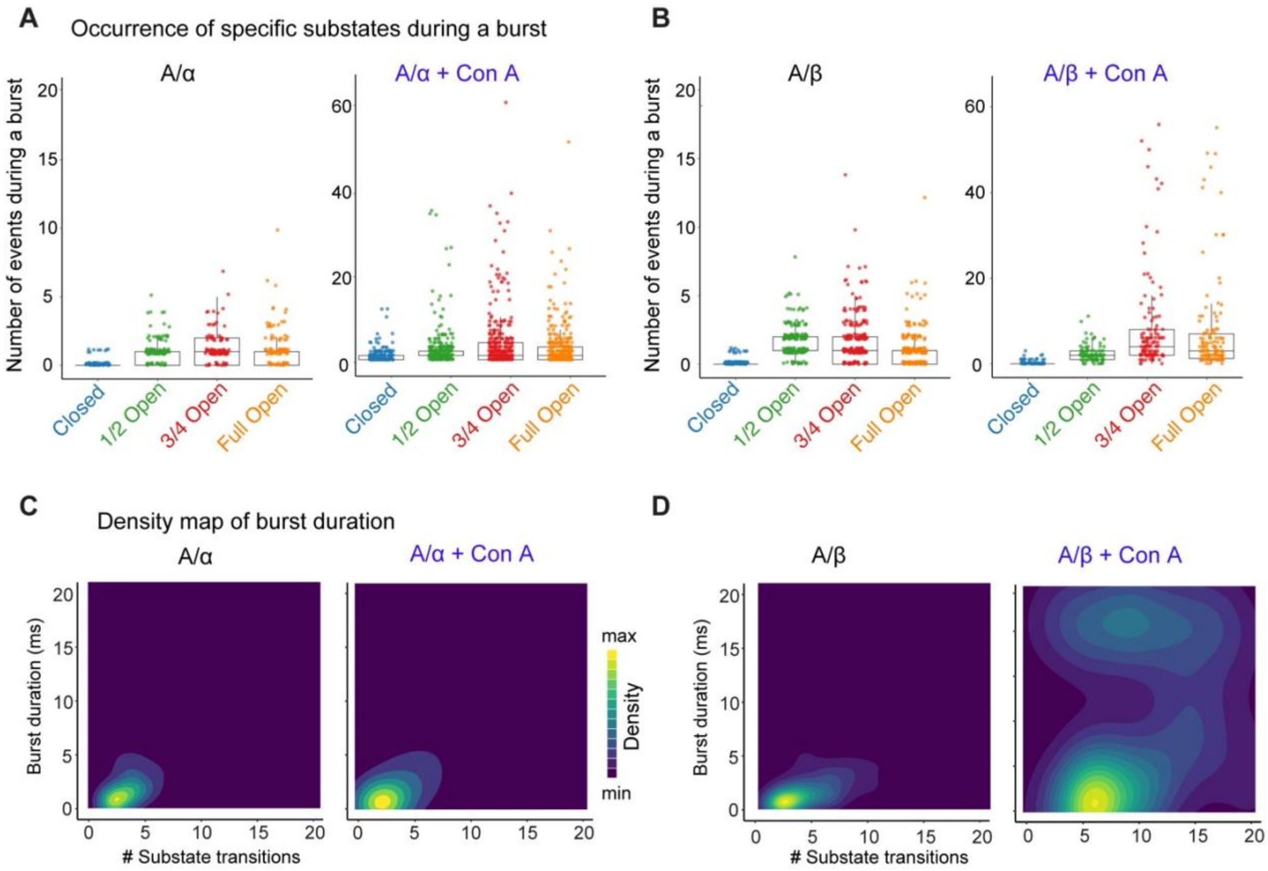

Supplemental figure S3. Distribution of substate transitions and burst characteristics. (**A-B**) Box plots indicating the number of specific events (substates occurrence) during a burst for A/α (**A**) and A/β (**B**) receptor channels before (left) and after (right) treatment with 0.6 mg/ml Con A for 10 min. The states closed, 1/2 open, 3/4 open, and fully open are color coded, as indicated. The boxes capture the interquartile range (IRQ) of each group's distribution of values; horizontal lines denote median values; vertical lines extend to 1.5x IQR. (**C-D**) Density plots illustrating the relationship between burst duration and the number of transitions among substates for the A/α and A/β receptor complexes as indicated, in the absence (left) of presence (right) of Con A.

bioRxiv preprint doi: https://doi.org/10.1101/2024.04.22.590603; this version posted April 26, 2024. The copyright holder for this preprint (which was not certified by peer review) is the author/funder. This article is a US Government work. It is not subject to copyright under 17 USC 105 and is also made available for use under a CC0 license.

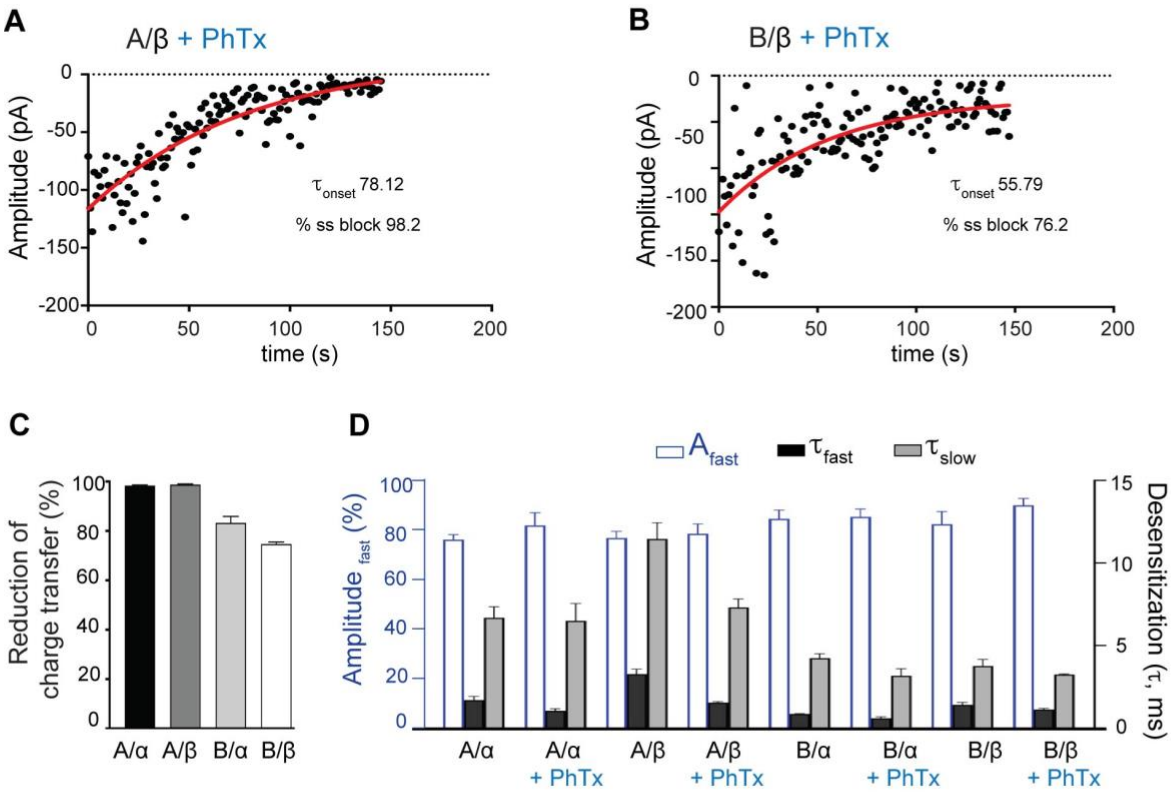

Supplemental figure S4. Slow onset of block by PhTx.

(**A-B**) Data points indicate the amplitude of sequential responses to 10 mM glutamate applied for 100 ms at intervals of 1 s for A/β and B/β. The amplitude variation is due to differences in the number of channels activated from trial to trial. Red lines show single exponential fits to the decay of the response to glutamate due to onset of block by 1 µM PhTx. (**C**) The extent of block by PhTX at equilibrium, estimated from the change in charge transfer with respect to control, where a value of 100% indicates complete block, with values A/α, 98.4 ± 0.23 % (n = 5); A/β, 98.8 ± 0.30 % (n = 5); B/α, 83.31 ± 2.53 % (n = 5); B/β, 74.70 ± 0.86 % (n = 5). (**D**) Fits to the decay of the response to 100 ms applications of 10 mM glutamate (fit with the sum of two exponentials) before and after application of PhTx, showing mean values for $\%A_{fast}$, τ_{fast}, and τ_{slow} for the indicated combinations of type-A and type-B complexes with Neto-α and Neto-β.
